# Comparison of organelle genomes between endangered mangrove plant *Dolichandrone spathacea* to terrestrial relative provides insights into its origin and adaptative evolution

**DOI:** 10.3389/fpls.2024.1442178

**Published:** 2024-09-23

**Authors:** Ying Zhang, Jingwen Zhang, Zewei Chen, Yanni Huang, Jiaxuan Liu, Yuqi Liu, Yong Yang, Xiang Jin, Yuchen Yang, Yiqing Chen

**Affiliations:** ^1^ Hainan Academy of Forestry, Hainan Mangrove Research Institute, Haikou, China; ^2^ Mangrove Rare and Endangered Species Protection and Utilization Engineering Technology Research Center, Zhanjiang Key Laboratory of Mangrove Ecosystem Protection and Restoration, Lingnan Normal University, Zhanjiang, China; ^3^ Ministry of Education Key Laboratory for Ecology of Tropical Islands, Key Laboratory of Tropical Animal and Plant Ecology of Hainan Province, College of Life Sciences, Hainan Normal University, Haikou, China; ^4^ State Key Laboratory of Biocontrol, School of Ecology, Sun Yat-sen University, Shenzhen, China

**Keywords:** mangrove associate, mitochondrial and chloroplast genomes, sequence divergence, splitting time, positive selection

## Abstract

*Dolichandrone spathacea* is a mangrove associate with high medicinal and ecological values. However, due to the dual-pressure of climate change and human activities, *D. spathacea* has become endangered in China. Moreover, misidentification between *D. spathacea* and its terrestrial relative *D. cauda-felina* poses further challenges to field protection and proper medicinal usage of *D. spathacea*. Thus, to address these problems, we sequenced and assembled mitochondrial (mt) and chloroplast (cp) genomes for both *D. spathacea* and *D. cauda-felina*. Comparative analysis revealed apparently different size and scaffold number between the two mt genomes, but a high similarity between the cp genomes. Eight regions with high sequence divergence were identified between the two cp genomes, which might be used for developing candidate DNA markers for distinguishing the two species. The splitting between *D. spathacea* and *D. cauda-felina* was inferred to occur at ~6.8 - 7.7 million years ago (Mya), which may be driven by the environment fluctuations in late Miocene. In the cp genome, 12 genes related to the expression of photosynthesis-associated proteins were detected with signatures of positive selection, which may contribute to the origin and evolutionary adaptation of *Dolichandrone* mangrove species. These new findings do not only enrich organelle genomic resources of *Dolichandrone* species, but also provide important genetic clues for improving the conservation and proper usage of endangered mangrove associate *D. spathacea*.

## Introduction

**Figure 1:**
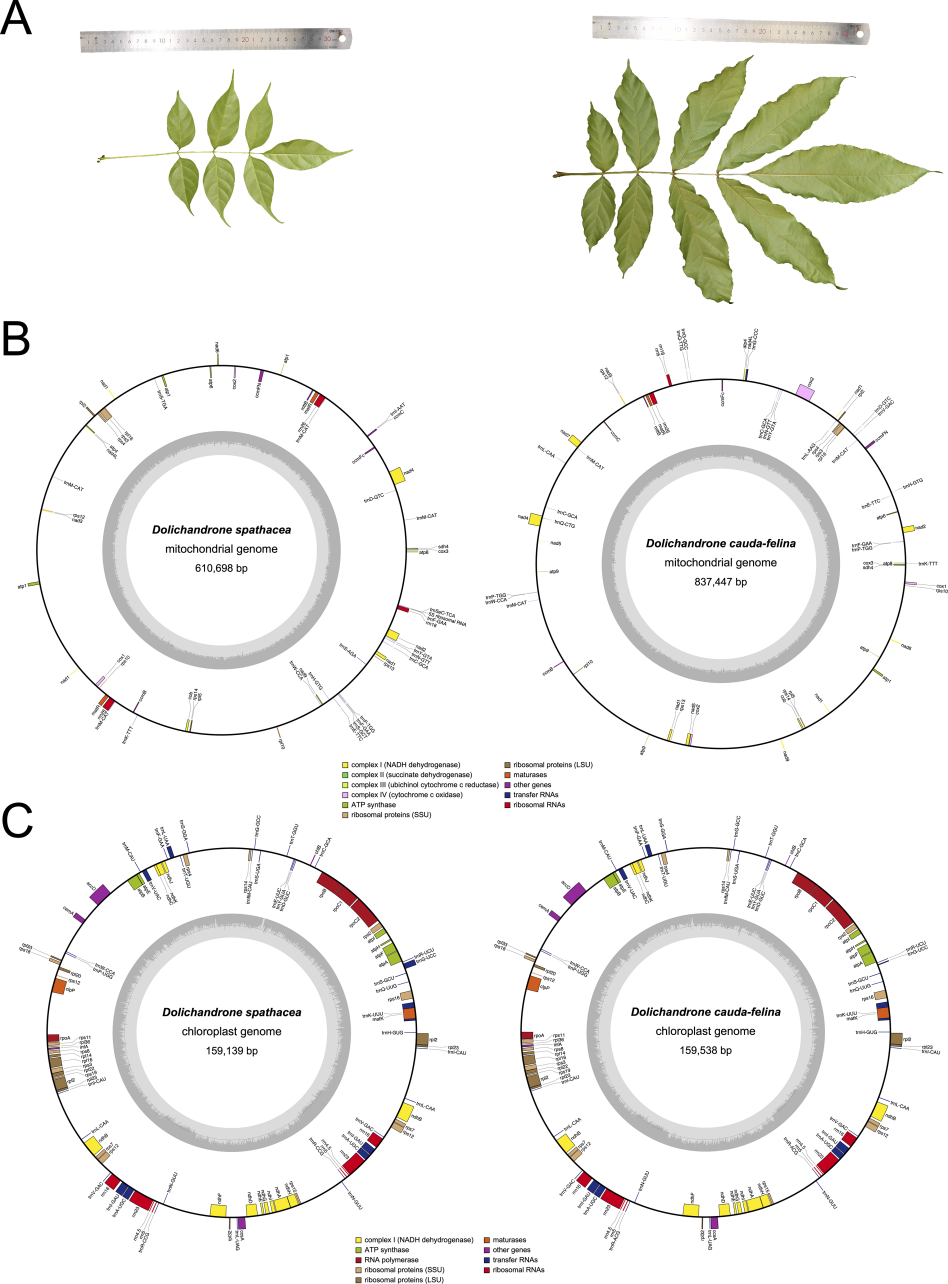
Leaf morphology and physical maps of mitochondrial and chloroplast genomes of *D. spathacea* and *D. cauda-felina*. **(A)** Photos illustrating morphological characters of *D. spathacea* (left panel) and *D. cauda-felina* leaf (right panel). **(B)** Maps of mitochondrial genomes of *D. spathacea* (left panel) and *D. cauda-felina* (right panel). Genomic features are shown facing outward (positive strand) and inward (negative strand) of mitochondrial genome represented as a circular molecule. The color key shows the functional class of the mt genes. The GC content is represented in the inner circle. **(C)** Maps of chloroplast genomes of *D. spathacea* (left panel) and *D. cauda-felina* (right panel). The genes transcribed clockwise are shown in the inner circle, and genes are transcribed counter-clockwise are shown in the outer circle. Genes are color-coded based on their functions. The genome coordinate and GC content are shown in the inner circle.

*Dolichandrone* (Bignoniaceae, Lamiales) consists of 12 species distributed in Africa and tropical Asia (Jackes, 2017). Of them, *D. spathace*, also known as Mangrove Trumpet Tree, is a mangrove associate that natively grows in coastal forests and waterlogged shoreline and estuary (Tomlinson, 2016). The importance of mangrove associates has been demonstrated in terms of their contributions in beach stability maintenance, carbon sequestration and mangrove habitat restoration (Mitra and Zaman, 2020). In addition to the ecological importance, *D. spathacea* also has medicinal values and has been utilized for the treatments of diabetes and cancers (Thao et al., 2021). However, in recent decades, mangrove forests have suffered an over 30% loss of natural distribution, and a subsequent loss of their ecological functions, under the dual-pressure of global climate change and human activities (Polidoro et al., 2010; Fan and Wang, 2017). In China, *D. spathacea* has been listed on the Conservation of the Key Protected Plants of Hainan Island (Zhang et al., 2021). Moreover, in the field, *D. spathacea* sometimes is difficult to be distinguish from its terrestrial relatives, for example, *D. cauda-felina*. One of the main differences between *D. spathacea* and *D. cauda-felina* is leaf size. Leaves and leaflets of *D. spathacea* are generally 20-30 cm and 5-10 cm in length, respectively, which are apparently shorter than those of *D. cauda-felina* (30-50 cm and 16-20 cm long, respectively) (**Figure 1A**). The mistaken recognition between close-related species may cause drug misuse and threatens the medication safety.

With the rapid development of high-throughput sequencing technologies, genome-wide researches open new avenues for the protection and proper usage of plants. Recent studies revealed that many true mangrove species displayed an extremely low genetic diversity and small effective population size, mirroring their particular vulnerability to future environmental fluctuations (Guo et al., 2018; He et al., 2022). Moreover, increased accumulation of genomic data can also facilitate the development of DNA markers, which is an effective solution for distinguishing close-related species. However, most previous studies mainly focused on the morphological and physiological specialization of mangrove associates (Wang et al., 2010; Quadros et al., 2021), and little attention has been paid to their genomic features, which leaves a knowledge gap.

In plants, chloroplast (cp) and mitochondria (mt) genomes have been widely used to investigate the evolutionary history of higher plants (Nizam et al., 2022). Both organelles are semi-autonomous that possess their own genetic materials separate from those in cell nucleus. Compared to the nuclear DNA, cp and mt genomes have smaller size and no sexual recombination (M. Wolfe et al., 1987; Salih et al., 2017), which make them much easier to be sequenced. Moreover, mt genomes have also been shown to possess many different characteristics from cp genomes, including smaller number of genes, lower mutation rate and larger structural variation (Wu et al., 2022; Wang et al., 2024). Recent studies have widely used organelle genomes to investigate the molecular evolution (Huang et al., 2020; Li et al., 2023). Compared to mt genome, the higher mutation rate and conserved structure of cp genome make it an ideal system for investigating phylogenetic relationships of different plant species, as well as developing molecular markers for discrimination of closely-related species (Mower and Vickrey, 2018; Fan et al., 2022; Nizam et al., 2022; Temel et al., 2022; Wei and Li, 2022). For mangrove plants, some sequence variations in cp loci were supposed to contribute to their adaptations to the harsh intertidal environments (Zhang et al., 2020; Temel et al., 2022; Tan et al., 2023). However, no current study has yet characterized the differences between the organelle genomes of *D. spathacea* and its terrestrial relatives of the same genus, restricting our understanding of the origin and adaptive evolution of *Dolichandrone* mangroves.

To address the gap, we sequenced and assembled mt and cp genomes of *D. spathacea* and its terrestrial congener *D. cauda-felina*. With the data, we aimed to (1) characterize sequence and structural features of organelle genomes of the two *Dolichandrone* species, (2) detect cp and mt genomic regions with high sequence variations, which can be used to design candidate molecular markers for distinguishing the two species, and (3) explore signature of positive selection within the organelle genomes that may contribute to the adaptive evolution of *D. spathacea*.

## Materials and methods

### Plant materials and DNA isolation

Seeds of *D. spathacea* and *D. cauda-felina* were collected from their natural habitats in Tielu Bay (18°14′26″N, 109°32′50″E) of Sanya, Hainan Island, China. The corresponding voucher specimens of *D. spathacea* and *D. cauda-felina* were deposited in the herbarium of Hainan Normal University (DS-001 and DC-001). Seeds were soaked in water for 24 h, and then planted into a plastic seed box with a medium composed of 60% sand and 40% soil, and germinated under a controlled a growth condition (14/10 h light/dark photoperiod, 80% humidity and 30**°**C). When the seedlings were six-month old with four true leaves, genomic DNA of young leaves was extracted using the DNA extraction kit (TransGen, China) following the manufactory’s instruction, and sequenced on an Illumina HiSeq platform (Illumina Inc., CA, USA) with 150 bp paired-end reads.

### Assembly and annotation of mt and cp organelle genomes

A total of 10.2 and 10.8 Gb of raw reads were produced for the two samples. FastQC software (http://www.bioinformatics.babraham.ac.uk/projects/fastqc/) was used to evaluate the quality of raw reads. The organelle genomes of *D. spathacea* and *D. cauda-felina* were assembled using SPAdes software v. 3.9.0 (Bankevich et al., 2012), and the assembled cp and mt scaffolds were then blasted against the sequences of the published cp genome of *Spathodea campanulata* (NC_049000) and mt genome of *Olea europaea* subsp. Europaea (LR743801.1) by BLASTn and Exonerate (Couture et al., 2009) with an e-value cutoff of 1e-10 and a protein similarity threshold of 70%. The matched scaffolds with high coverage were retained, interatively extended and reconstructed using Paired-Read Iterative Contig Extension (PRICE) (Ruby et al., 2013) and MITObim (Hahn et al., 2013). Bowtie2 (Langmead and Salzberg, 2012) was used to aligned the reads to the iteratively assembled outputs, and the aligned reads were reassembled using SPAdes. This process was repeated until a circular genome was obtained. Genes were predicted in each cp genome using online annotation tool DOGMA (Wyman et al., 2004), and the genes encoding transfer RNAs (tRNAs) and ribosomal RNAs (rRNAs) were further verified using tRNAscan-SE v. 2.0 software (Chan et al., 2021) and RNAmmer Server v. 1.2 (http://www.cbs.dtu.dk/services/RNAmmer/), respectively. The structural features of the cp genomes were illustrated by OGDRAW software v.1.3.1 (Greiner et al., 2019). Each mt genome was manually annotated using MFannot (Beier et al., 2017) and Geneious (Kearse et al., 2012). Moreover, for each species, the sequences of the two organelle genomes were aligned and compared using Mauve software (Darling et al., 2004) with default parameters, to detect putative cp-derived sequences in the corresponding mt genome.

### Analyses of repeats, codon usage and putative RNA editing sites

Repeat sequences were analyzed for each cp and mt genome using REPuter (Kurtz et al., 2001) with hamming distance = 3, maximum computed repeats = 5,000, and minimal repeat size = 50. The online tool MISA-web (Beier et al., 2017) was used to identify simple sequence repeats (SSRs), with minimum repetition numbers of mono-, di-, tri-, tetra-, penta-, and hexanucleotides set as 10, 5, 4, 3, 3, and 3, respectively. Dispersed and tandem repeats were identified by Tandem Repeats Finder (Benson, 1999). Transposable elements (TEs) were annotated in the mt genomes using CENSOR program with default parameters, and their locations in genome were visualized with Circos (Kohany et al., 2006). For each organelle genome, CodonW software (Liu et al., 2018) was used to characterize codon usages of each non-repetitive coding sequence (CDS) with a length > 300 bp. RNA editing sites were scanned in the protein-coding genes (PCGs) of each cp genome using PREP suite with a cutoff value (C) of 0.8 (Mower, 2009).

### Sequence variability analysis

Sequence variability between the two *Dolichandrone* cp genomes was visualized using mVISTA program (Frazer et al., 2004), and cp genome of *D. cauda-felina* was used as the reference. To further investigate the highly variable regions, the two cp genomes were aligned using the MAFFT software (Katoh and Standley, 2016), and nucleotide divergence (Pi) was calculated using DnaSP v. 6.12.03 with the length of sliding window set to 600 bp and the step size set to 200 bp (Librado and Rozas, 2009).

### Phylogeny reconstruction and divergence time estimation for Bignoniaceae species

To infer the origin time of the mangrove species *D. spathacea*, a phylogenetic analysis was first performed using the cp genomes of the two *Dolichandrone* species and nine other Bignoniaceae species. *Aloysia citrodora* from Verbenaceae was used as the outgroup. All the published cp genomes used for this analysis were downloaded from the GenBank database (their accession numbers were listed in **Supplementary Table S13**). The sequences were aligned using MAFFT software (Kim et al., 2023) with default parameter setting. Phylogenetic tree was constructed by a maximum likelihood (ML) approach in RAxML-HPC v.8.2.12 (Stamatakis, 2014), where the GTR + GAMMA + I substitution model was selected and the bootstrap replicate was set to 1,000.

Divergence time among the 12 tested species was estimated using BEAST v.2.5 (Bouckaert et al., 2019). The input files were formatted with BEAUti interface (Drummond et al., 2012), according to the alignment result of MAFFT. The topology of the tree prior of the 12 species was set based on their phylogenetic relationships inferred by the ML analysis. Calibrated Yule Model tree prior and strict clock model were used, and Gamma category count, iterations of Markov chain Monte Carlo (MCMC), and pre-burnin was set as 4, 1,000,000 and 0, respectively. According to the fossil record, a calibration of 50 million year ago (Mya), with a 95% credit interval (CI) of 45 to 55 Mya, was set at the root node of Bignoniaceae species and the outgroup *A. citrodora* (Lohmann et al., 2013; Asaf et al., 2020). The estimation results of BEAST were summarized onto a maximum clade credibility tree using TreeAnnotator v.2.7.4 of BEAST.

### Positive selection analysis between two *Dolichandrone* species

Homologous cp and mt PCG pairs were determined between the two *Dolichandrone* species by Blastn based on their similarities in protein sequences, and were aligned by ParaAT2.0 (Zhang et al., 2012). Then, the nonsynonymous substitution (Ka) rate, synonymous substitution (Ks) rate and their ratio (Ka/Ks) were computed for these genes using Ka/Ks Calculator v.2.0 (Wang et al., 2010). For each tested gene, a Ka/Ks ratio < 1 indicates that its synonymous substitutions were more common than nonsynonymous ones, and it was under purifying selection; Ka/Ks ratio = 1 suggests neutral selection; and Ka/Ks ratio >1 implies that it might has been positively selected (Hurst, 2002; Ramundo et al., 2013). We further conducted positive selection analyses under the phylogenetic context using the branch-site model of PAML (Yang, 2007). A total of 58 polymorphic cp PCGs commonly shared in all the 12 species were examined. *D. spathacea* and *D. cauda-felina* were set as the “foreground branch”, respectively, and all other branches in the phylogenetic tree were set as “background branches”. The genes with ω > 1 and a p-value of likelihood ratio test (LTR) < 0.05 were considered to be under positive selection.

## Results

### Structural features of mitochondrial genomes of *D. spathacea* and *D. cauda-felina*

The assembled mt genome of *D. spathacea* contained two scaffolds of 610,698 bp (DS1, GC content of 44.94%) and 59,125 bp (DS2, GC content of 44.40%) in length, respectively, while its terrestrial relative *D. cauda-felina* had only one scaffold of 837,447 bp (GC content of 44.79%) (**Figure 1B**; **Table 1**). A total of 38 PCGs and 23 tRNA- and 4 rRNA-coding genes annotated in *D. spathacea*, and 38 PCGs, 22 tRNA-coding genes and 3 rRNA-coding genes were detected in *D. cauda-felina* mt genome. Genes *trnG-CCC, trnL-AAG, trnL-CAA* and *trnV-GAC* are exclusively found in *D. cauda-felina* mt genome, while *trnI-AAT, trnS-AGA, trnS-TGA, trnS-GCT,* and *trnSeC-TCA* are exclusively observed in *D. spathacea* (**Supplementary Table S1**). Moreover, there were two copies of rrn26 in *D. spathacea* mt genome, but only one copy was in *D. cauda-felina.*

**Table 1:** General features of mitochondrial and chloroplast genomes of *D. spathacea* and *D. cauda-felina*.

	Mitochondria			Chloroplast	
	*D. cauda-felina*	*D. spathacea*		*D. cauda-felina*	*D. spathacea*
		scaffold 1 (DS1)	scaffold 2 (DS2)		
Genome size	837,447 bp	610,698 bp	59,125 bp	159,538 bp	159,139 bp
GC content	44.79%	44.94%	44.40%	38.2%	37.9%
Protein-coding genes^*^	38	36	2	87	88
tRNA-coding genes^*^	22	20	3	37	37
rRNA-coding genes	3	4	0	8	8
Coding genes with introns	7	6	1	10	10
tRNA with introns	–	–	–	3	3
Repeat sequences	3.25%	2.23%	–	2.26%	2.02%
Cp-derived	14%	6%	–	–	–

^*^The number presented here is the total number of genes in each genome.

**Figure 2:**
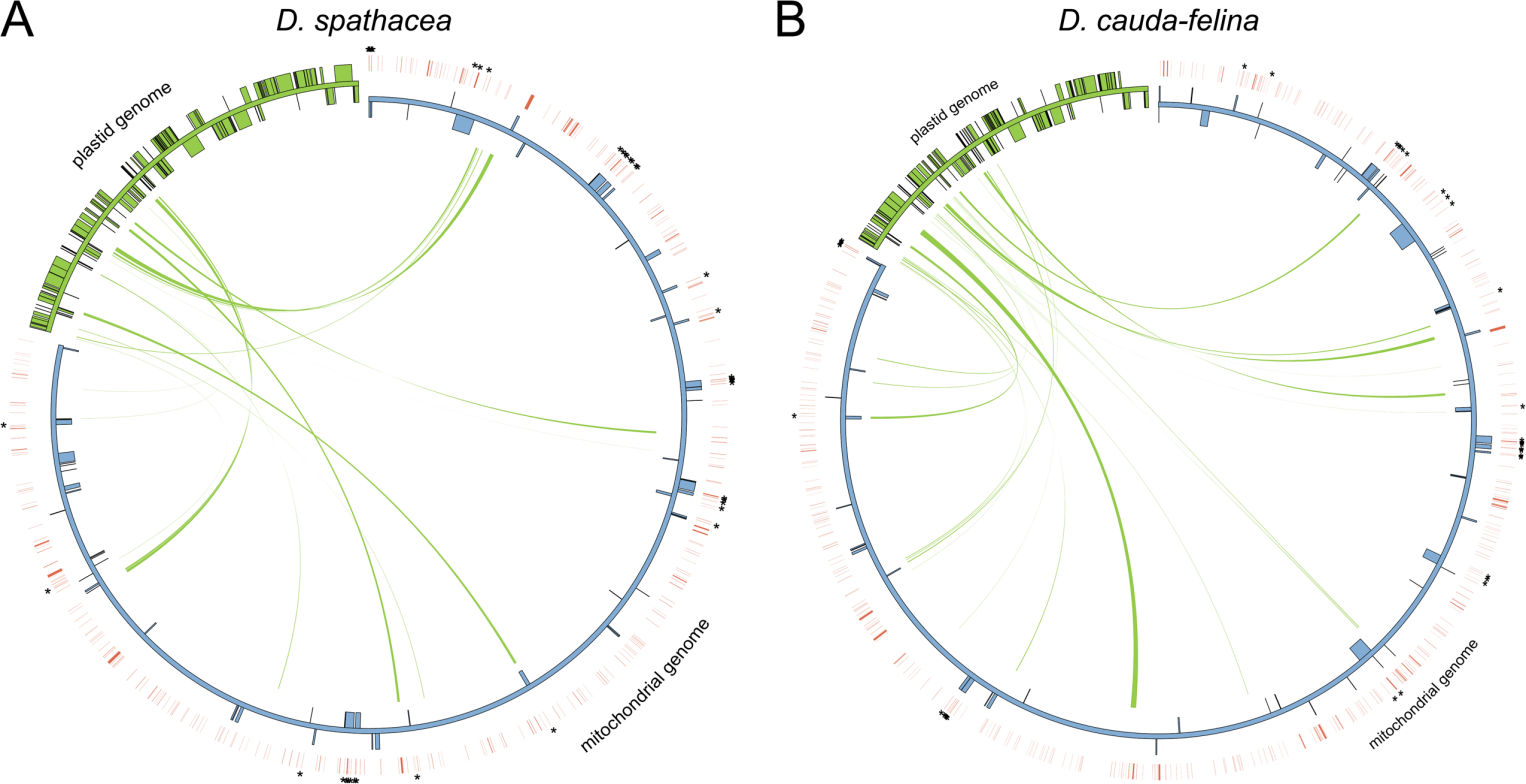
Schematic representation of transfers of chloroplast (cp) DNA and transposable elements (TEs) into the mitochondrial (mt) genomes of *D. spathacea* and *D. cauda-felina*. **(A)** Cp DNA transferred into the mt genome of *D. spathacea*. **(B)** Cp DNA transferred into the mt genome of *D. cauda-felina*. In each panel, green lines within the circle show the regions of the plastid genome that have been inserted into different locations of the mt genome. Blue and green boxes on the inside and outside of maps present mt and cp genes transcribed clockwise and counter clockwise directions. Red lines outside of the two genomes present positions of TEs, and the TEs inserting into genic regions are highlighted by asterisks.

There were 8,362 bp and 12,259 bp of repetitive DNA in the mt genomes of *D. spathacea* and *D. cauda-felina*, respectively, ranged from 37 to 1,643 bp and 40 to 3,036 bp in length (**Supplementary Table S2**). The percentage of repeated structure of *D. spathacea* was apparently lower than that in *D. cauda-felina* (**Table 1**). Of them, 41 and 52 SSRs were detected in *D. spathacea* and *D. cauda-felina*, respectively (**Supplementary Table S3**). In addition, 11 and 13 abundant tandem repeats and 27,834 bp (4.6%) and 36,629 bp (4.4%) of TEs were observed in *D. spathacea* and *D. cauda-felina* mt genomes (**Supplementary Tables S4, S5**). Most of the TEs were copia- and gypsy-like retrotransposons, and the majority of TEs were presented in intergenic regions, with only 1,496 bp and 1,674 bp elements inserted into genic regions (**Supplementary Table S6**). A total of 26 and 18 fragments in *D. cauda-felina* and *D. spathacea* mt genomes were identified to be homologous to the corresponding cp genome sequences, which accounted for ∼1.5% and ∼1.4% of the two mt genomes, respectively (**Figure 2**).

### Structural features of chloroplast genomes of *D. spathacea* and *D. cauda-felina*

The sizes of the *D. spathacea* and *D. cauda-felina* cp genomes were 159,139 bp and 159,538 bp, respectively (**Table 1**). Both cp genomes consisted of a single circular molecule with the same quadripartite structure: a large single copy region (LSC) and a small single copy region (SSC), separated by two copies of inverted repeats (IRs) (**Figure 1C**). In the cp genomes of *D. spathacea* and *D. cauda-felina*, 81 and 80 PCGs, 30 tRNA-coding and 8 rRNA-coding genes were observed, respectively, where seven PCGs and seven tRNA-coding genes have two copies in both species. Gene *ycf12* is exclusively in *D. spathacea* (**Figure 1C**; **Table 1**, **Supplementary Table S7**). The average GC contents of the two cp genomes were 37.9% (*D. spathacea*) and 38.2% (*D. cauda-felina*), which were obviously lower than the corresponding mt genomes (**Table 1**). In the cp genomes of *D. spathacea* and *D. cauda-felina*, 44 and 34 SSRs and 94 and 75 abundant tandem repeats were detected, respectively (**Supplementary Tables S5, S8**).

### Codon usage and putative RNA editing sites in mitochondrial and chloroplast protein-coding genes

**Figure 3:**
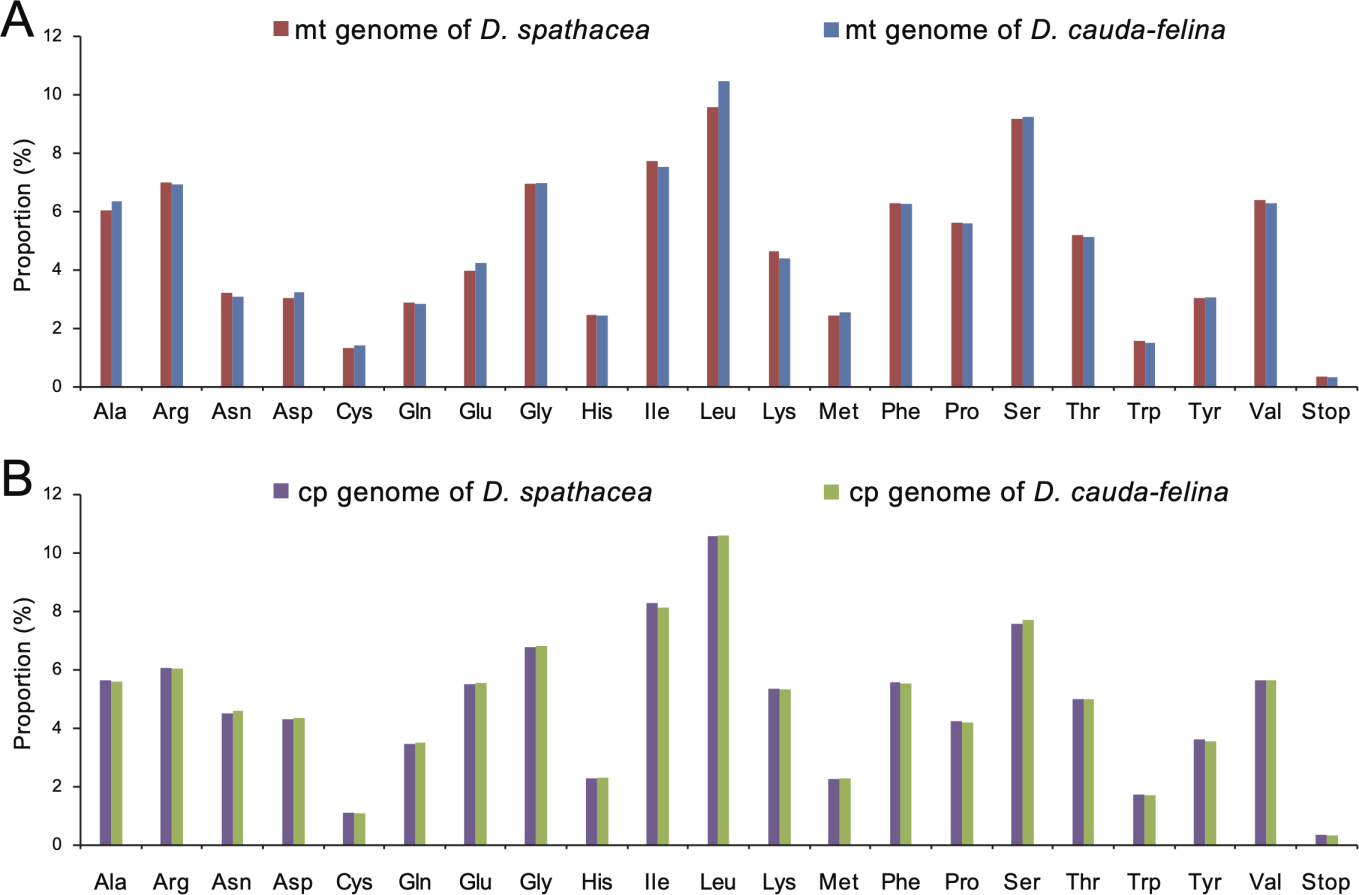
Amino acid usage frequencies in the organelle genomes of *D. spathacea* and *D. cauda-felina*. **(A)** Amino acid usage frequencies in the mitochondrial genomes of *D. spathacea* (red) and *D. cauda-felina* (blue). **(B)** Amino acid usage frequencies in the chloroplast genomes of *D. spathacea* (purple) and *D. cauda-felina* (green).

Codon usage frequency and relative synonymous codon usage (RSCU) were analyzed for each organelle genome. A total of 8,800 and 10,231 codons were identified in the PCGs of the mt genomes of *D. spathacea* and *D. cauda-felina*, and 23,006 and 23,108 codons were in the two cp genomes. In all the four organelle genomes, leucine (Leu) was the most frequently used amino acid residues, with a frequency of 9.56% to 10.58%, followed by serine (Ser) at 7.70% to 9.26% and isoleucine (Ile) at 7.74% to 8.28%. In contrast, cysteine (Cys) was of the lowest abundance, with a proportion of 1.08% to 1.43% (**Figure 3**; **Supplementary Table S9**). However, the other 17 commonly used codons displayed slightly different usage frequencies among the four organelle genomes (**Figure 3**). Most of the A/U-ending codons in both cp and mt genomes had RSCU >1, suggesting a more frequent usage of A or U at the third codon position. In contrast, most of the G/C-ending codons had RSCU values <1, except methionine (Met) and tryptophan (Trp) which exhibited an RSCU =1 (**Supplementary Table S9**). In total, 269 and 308 potential RNA editing sites were detected in 28 and 31 mt PCGs of *D. spathacea* and *D. cauda-felina*, respectively, and 45 and 42 RNA editing sites were observed in 18 and 19 PCGs of two cp genomes. Most conversions at the RNA editing sites were from serine (S) to leucine (L), and genes *ndhB* and *ndhD* genes consisted of the largest numbers of RNA editing sites (**Supplementary Table S10**).

### Sequence divergence between the chloroplast genomes of two *Dolichandrone* species

**Figure 4:**
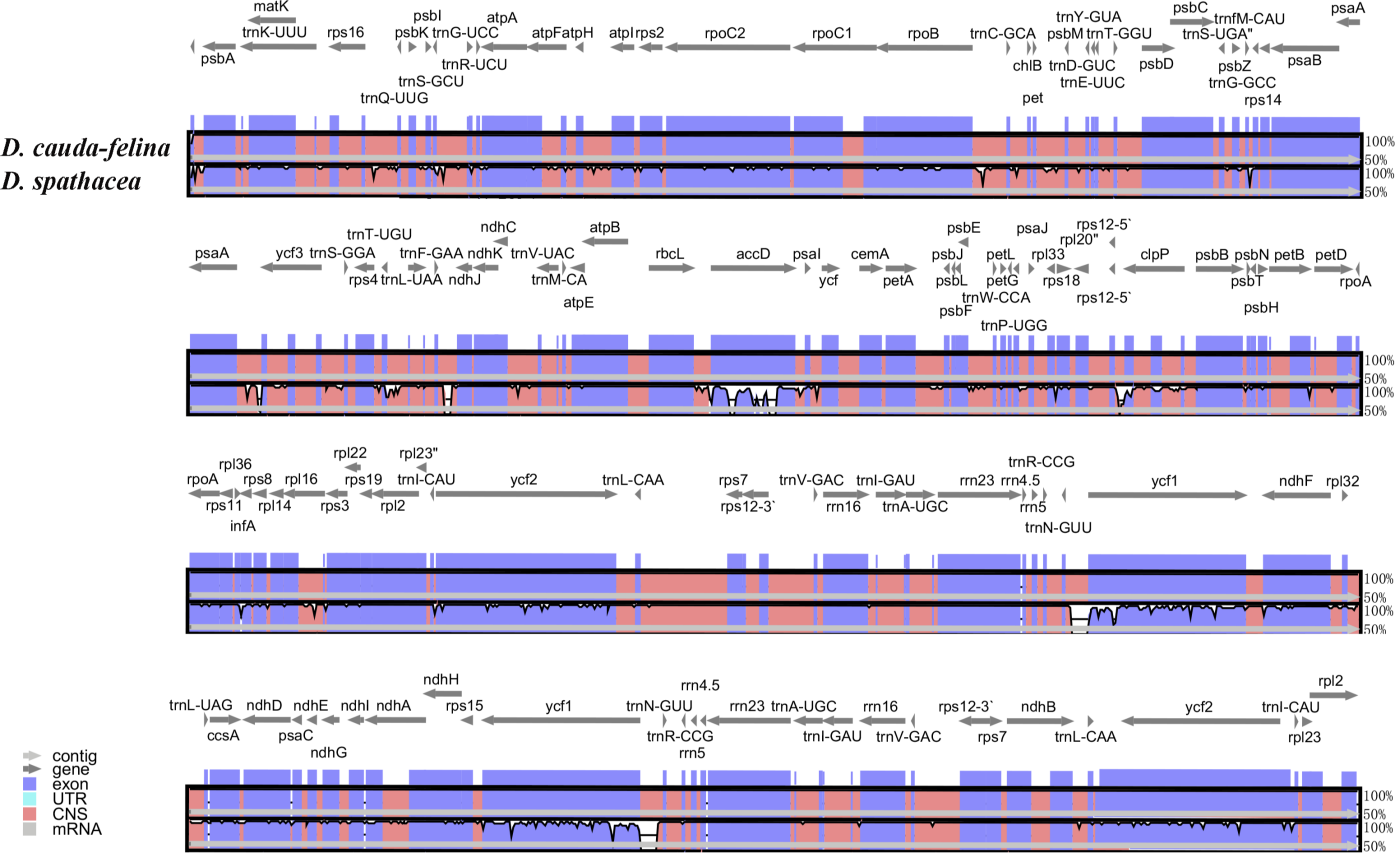
Sequence alignment of two *Dolichandrone* chloroplast genomes, with *D. cauda-felina* as the reference. The y-axis indicates the percent identity between 50% and 100%. Genes and contigs are presented by dark grey and grey arrows. Different colored represent different genome elements exons (purple), untranslated regions (UTRs) (turquoise) and conserved noncoding sequences (CNS) (red).

**Figure 5:**
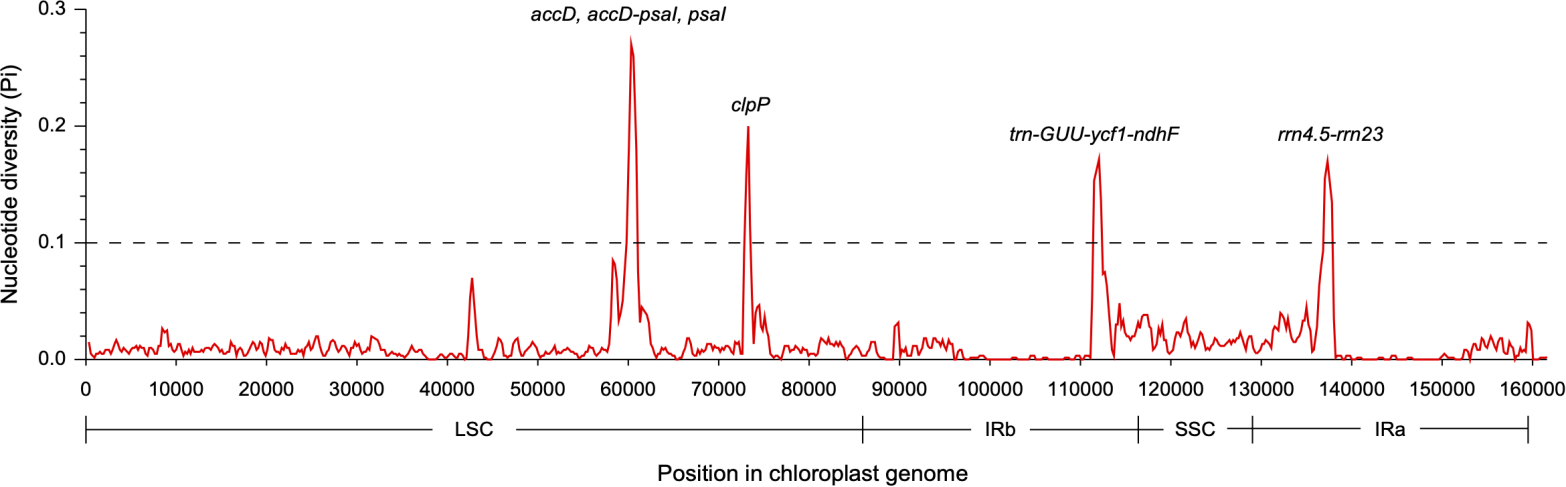
Nucleotide diversity across the chloroplast genomes of two *Dolichandrone* species. Midpoint is used to represent each sliding window.

The coding regions of the two *Dolichandrone* cp genomes were almost identical, except for *accD, ycf1, rpoB-trnC-GCA, psaA-ycf3, trnF-GAA-ndhJ, rps12-clpP* and *trnN-GUU-ycf1* (**Figure 4**). By the contrary, the noncoding region displayed a higher degree of sequence variation between the two species (**Figure 4**). IR regions were more similar than LSC and SSC regions, suggesting its high conservation throughout evolution (**Figure 4**). Between the two *Dolichandrone* cp genomes, Pi values ranged from 0 to 0.270, and seven hypervariable regions with Pi > 0.1 were identified between *D. spathacea* and *D. cauda-felina* (*accD, accD-psaI, psaI, clpP, trn-GUU-ycf1-ndhF, rrn4.5-rrn23* and *rrn23*) (**Figure 5**; **Supplementary Table S11**).

### Phylogenetic relationships and divergence time of Bignoniaceae species

**Figure 6:**
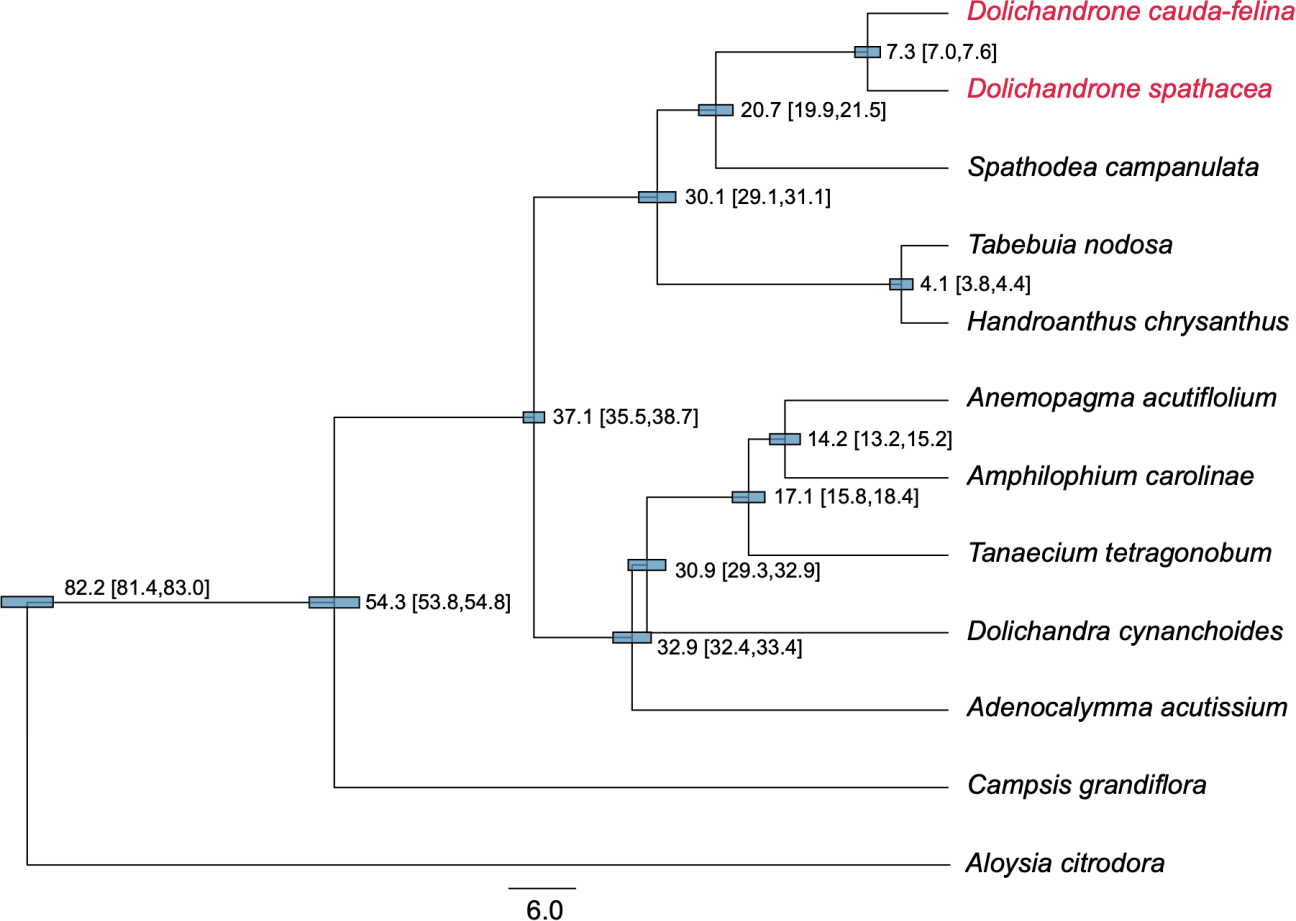
Phylogenetic tree and divergent time of 11 Bignoniaceae species. The tree was constructed based in 46 protein-coding genes of cp genomes using BEAST software program. *Aloysia citrodora* from Verbenaceae was used as the outgroup. The estimated divergent time and 95% highest posterior density (HPD) interval (in millions of years (Mya)) are presented at each node. The length of blue bars corresponds to the 95% HPD interval of each node. Two *Dolichandrone* species are highlighted in red.

Compared to plant mt genome, cp genome has a higher synonymous substitution rate, thus is more suitable for reconstructing phylogenetic relationships among close-related plant taxa (Wolfe et al., 1987; Fan and Ma, 2022). In this study, we reconstructed a phylogenetic tree for 11 Bignoniaceae species using their cp genomes to infer the origin time of *D. spathacea.* The topology of the ML tree was highly consistent with our prior knowledge (Olmstead et al., 2009) (**Figure 6**). The Bignoniaceae species were clustered into three major clades, where *Campsis grandiflora* from Tecomeae first diverged in the family (approximate divergent time: 54.3 Mya). The species of Bignonieae tribe and Crescentiina group were supposed to split at 37.1 Mya (95% highest posterior density (HPD) interval: 35.5 - 38.7 Mya). In Crescentiina group, the species from Tabebuia alliance (*Tabebuia nodosa* and *Handroanthus chrysanthus*) and Paleotropical clade fell into different clusters, and within the Paleotropical clade, *Spathodea campanulate* was sister to the two *Dolichandeone* species. The splitting time between *Dolichandeone* species and *S. campanulata* was estimated to be 20.7 Mya (95% HPD interval: 19.9 - 21.5 Mya), and the divergence of mangrove species *D. spathaecea* and its terrestrial relatives *D. cauda-felina* was supposed to occur at 7.3 Mya (95% HPD interval: 7.0 - 7.6 Mya) (**Figure 6**).

### Positively selected genes in *Dolichandrone* species

We then computed the Ka rate, Ks rate and their ratio (Ka/Ks) for 79 homologous cp PCGs and 33 homologous mt PCGs between the two *Dolichandrone* species (**Supplementary Table S12**). All the mt PCGs were recorded to be under purifying selection (Ka/Ks < 1.0), while the Ka/Ks ratio of 12 cp genes (*accD, atpF, clpP, rpoB, rpoC2, rps12, rps15, rps18, rps2, rps8, ycf1* and *ycf2*) were >1.0, suggesting the signals of positive selection. Positive selection analyses were further performed based on the phylogeny of the 11 Bignoniaceae species and an outgroup. The results showed that, in *D. spathacea*, gene *ccsA* was supposed be under positive selection, while *psbB* and *ccsA* were considered to be positively selected in *D. cauda-felina*.

## Discussion

A comprehensive investigation to the origination and evolutionary history of endangered mangrove species *D. spathacea* can improve our understanding of their adaptation to coastal environment and assist their protection and proper usages. A previous study based on whole genome sequencing suggested the splitting between *Dolichandeone* and *Tabebuia* species occurred at ~32.29 Mya (He et al., 2022). However, the exact origin time of mangrove species *D. spathacea* within *Dolichandeone* is still unknown. In this study, we reconstructed the phylogeny of Bignoniaceae species based on their cp genomes. The result revealed that the splitting time of the species from Tabebuia alliance and Paleotropical clade was inferred to be ~29.7 - 31.3 Mya, which is highly consistent with the previous estimation (~32.29 Mya) (He et al., 2022), suggesting the reliability of our analysis. More importantly, the divergence between *D. cauda-felina* and *D. spathacea* was supposed to occur at approximately 6.8 - 7.7 Mya (the Early Pliocene; **Figure 6**). Most of the current species of *Dolichandrone*, as well as the fossil species *D. wuhanensis*, are distributed in warm and humid areas in tropical or subtropical Asia and West Pacific Region (Qi et al., 1997). In late Miocene, global temperature, sea level and CO_2_ concentration experienced substantially fluctuation (Hansen et al., 2013), which might convert some rainforest habitats into coastal wetland, and provide opportunities for inland *Dolichandrone* ancestors to enter the intertidal environment and evolve into mangrove plants.

We further characterized the differences in the mt and cp genomes between the two *Dolichandrone* species. The mt genome assembly of *D. spathacea* consists of two scaffolds, while the terrestrial species *D. cauda-felina* contains only one scaffold (**Figure 1B**; **Table 1**). For the mt genomes of land plants, a large variation of the physical structures has been observed, such as single circular molecules, multiple circular structures, linear arrangements and branched forms (Li et al., 2021). Similar to *D. spathacea*, both the mt genomes of *Amorphophallus albus* (Shan et al., 2023) and *Hemerocallis citrina* (Zhang et al., 2022) present a multiple chromosomal structure. The complicated structural configurations make the mt genomes of most land plants difficult to assemble accurately (Wang et al., 2024). Furthermore, the mt genome size of *D. spathacea* (610,698 bp and 59,125 bp for the two scaffolds) is substantially smaller than its terrestrial relative *D. cauda-felina* (**Figures 1B, C**). Size variations in plant mt genomes are affected by many factors, such as increase in repeats, movement of foreign sequences, and the acquisition or loss of large intrageneric segments (Wu et al., 2022). Compared to its terrestrial relative, the percentage of repeated structure in *D. spathacea* was significantly lower. Moreover, we observed a lower number of plastid-like fragments in *D. spathacea* mt genome than that of *D. cauda-felina* (**Supplementary Figure S1**), suggesting the transfer of sequences from the cp genome might also play a role in affecting the size of the mt genome in angiosperms (Saina et al., 2018).

Unlike mt genomes, the cp genomes of the two *Dolichandrone* species are much smaller and more conserved in terms of both sequence and genomic structure (**Figure 1C**). In *D. spathacea*, we observed a loss of gene *ycf12* from the cp genome, although it existed in many angiosperms (Kashino et al., 2007). Similar fragment loss was also found in other higher plants such as *Trentepohlia odorata*, *Cephaleuros parasiticus* and *C. karstenii* (Fang et al., 2021). Contraction of the IR region was a common phenomenon during plant evolution (Huang et al., 2014), and it might be one of the main reasons for the smaller cp genome size of *D. spathacea*. In addition, the percentage of repeated structure in *D. spathacea* was also less than that in *D. cauda-felina*. The majority of sequence variations between the two *Dolichandrone* cp genomes occurred in noncoding regions, which is a frequent phenomenon in higher plants (Wicke et al., 2011; Fan and Ma, 2022; Jia et al., 2023). In coding region, four mutation hotspot regions were identified: *accD*, *clpP*, *psaI* and *ycf1* (**Figure 5**). These hypervaraibale regions can be used for designing specific genetic markers for phylogenetic and population genetic studies of *Dolichandrone* species. For example, the high nucleotide diversity of *ycf1* was also observed among other congeners, which makes it to be an ideal plastid DNA barcode for land plants (Dong et al., 2015; Kim et al., 2023). A benchmarking of 490 samples from 420 tree species showcased the outperformance of *ycf1* in distinguishing different species with up to 26.8% improvement in discriminating rate over the other two commonly-used cp markers, *matK* and *rbcL* (Dong et al., 2015). Our results further demonstrating the advantages of *ycf1* in phylogenetic application at a species-level resolution.

The codon preference assessment showed that, in both *Dolichandrone* species, cp genes exhibit a strong codon usage bias of A/U base at the third codon position (RSCU > 1; **Supplementary Table S9**). The same conclusion was also reached in the study of in other angiosperms (Huang et al., 2014; Li et al., 2019; Liang et al., 2022). It is a combined consequence of genomic mutation, genetic drift and natural selection (Geng et al., 2022). Codon usage bias can affect the efficiency of gene transcription and translation, and a comprehensive understanding of codon preference is beneficial for exploring evolutionary relationships among species and providing theoretic guidance for chloroplast genetic engineering and molecular breeding (Li et al., 2019; Geng et al., 2022; Wang et al., 2022b). Moreover, in both *Dolichandrone* species, leucine and serine are the highest abundant among the chloroplast amino acids, while cysteine is of the lowest amount. It is also similar to previous studies on other land plants (Yang et al., 2018; Wang et al., 2022a), which might mirror a specific correlation between codon usage bias and amino acid composition.

Genes linked to a specific environment are typically believed to be undergoing positive selection, and this has been utilized to identify genes related to environmental adaptation (Scobeyeva et al., 2021). In this study, the Ka/Ks values of all the mt loci between the two *Dolichandrone* species were less than 1.0. In the cp genomes, 12 loci were identified with the signals of positive selection (Ka/Ks > 1.0). Of them, *rps2*, *rps8*, *rps12*, *rps15* and *rps18* are the coding genes for ribosomal protein smaller (RPS) subunits. The mutations in these genes may influence the translation accuracy and efficiency of chloroplast proteins, and thus affect plant growth and development (Ramundo et al., 2013; Tiller and Bock, 2014; Schmid et al., 2024). Not only cp genome, He et al. also showed a high level of convergence in amino acid usage in nuclear genomes of different mangrove species, which are largely distinct from their terrestrial non-mangrove relatives (He et al., 2020). It might be because all the mangrove plants face the similar ecological pressure from the extremely intertidal habitats, and the specific amino acid composition can assist to cope with the environmental challenges.

## Conclusions

In this study, we assembled and characterized the complete cp and mt genomes of the endangered mangrove species *D. spathacea* and its terrestrial relative *D. cauda-felina*. Although structure and sequences of both organelle genomes were apparently similar between the two species, some genetic-variant hotspots were identified in cp genome, providing informative markers for investigating the origin and evolutionary history of mangrove species in this genus. Phylogenetic analysis revealed a divergence between *D. spathacea* and its terrestrial relative at 6.8 - 7.7 Mya, where the environment fluctuations in late Miocene might be the plausible driving factor. In the cp genome, 12 genes related to the expression of photosynthesis-associated proteins were subject to positive selection, which may contribute to the different environmental adaptations between *Dolichandrone* mangrove and non-mangrove plants. These new findings do not only offer reference data for cp and mt genomes of *Dolichandrone* species, but also provide a better understanding of the origin and adaptative evolution of the endangered mangrove species *D. spathacea*.

## Data Availability

The mitochondrial and chloroplast genomes of D. spathacea and D. cauda-felina were deposited in the NCBI Sequence Read Archive (SRA) with accession number of MW432176-MW432180.
